# Supplementary material for: Identification and functional analysis of non-coding regulatory small RNA FenSr3 in Bacillus amyloliquefaciens LPB-18
Source: PeerJ. 2023 May 15;11:e15236. doi: 10.7717/peerj.15236 (PMC10194069; doi:10.7717/peerj.15236)
Supplement: Supplemental Information 4 [file peerj-11-15236-s004.zip › KO/CK-vs-T1_map/map04977.html]

KEGG PATHWAY: Vitamin digestion and absorption - Reference pathway


|  |  |
| --- | --- |
| **Vitamin digestion and absorption - Reference pathway** |  |

[
Pathway menu
| Organism menu
| Pathway entry
| Show description
| User data mapping
]

Vitamins are a diverse and chemically unrelated group of organic substances that share a common feature of being essential for normal health and well-being. They catalyze numerous biochemical reactions. Because humans and other mammals cannot synthesize these compounds (except for some synthesis of niacin), they must obtain them from exogenous sources via intestinal absorption. Vitamins are classified based on their solubility in water or fat. Most of the water-soluble vitamins are transported across the small intestinal membrane by carrier-mediated mechanisms, but vitamin B12, cobalamin, is transported by a receptor-mediated mechanism. Intestinal absorption of fat-soluble vitamins requires all of the processes needed for fat absorption. After digestion, these vitamins and the products of pancreatic hydrolysis of triglycerides (TG) are emulsified by bile salts to form mixed micelles which are taken up by intestinal enterocytes and incorporated into chylomicrons (CM). CM are then secreted into the lymphatic system, and finally moves into the plasma.

|  |  |  |
| --- | --- | --- |
| Reference pathway | 184% 150% 122% 100% 82% 67% 55% | 图片下载 |
